# Stereotypes about surgeon warmth and competence: The role of surgeon gender

**DOI:** 10.1371/journal.pone.0211890

**Published:** 2019-02-27

**Authors:** Claire E. Ashton-James, Joshua M. Tybur, Verena Grießer, Daniel Costa

**Affiliations:** 1 Pain Management Research Institute, Faculty of Medicine and Health, The University of Sydney, Sydney, Australia; 2 Department of Experimental and Applied Psychology, VU University Amsterdam, Amsterdam, The Netherlands; 3 Department of Psychology, Ludwig Maximilian University Munich, Munich, Germany; University of Alabama at Birmingham, UNITED STATES

## Abstract

Past research indicates that patient perceptions of surgeon warmth and competence influence treatment expectancies and satisfaction with treatment outcomes. Stereotypes have a powerful impact on impression formation. The present research explores stereotypes about surgeon warmth and competence and investigates the extent to which surgeon gender influences perceptions of female and male surgeons. A between-subjects experiment was conducted online using crowdsourcing technology to derive a representative sample from the general population. Four hundred and fifteen participants were randomly assigned to evaluate the warmth and competence of males, females, surgeons, male surgeons, or female surgeons, using validated measures. Planned contrasts revealed that as a group, surgeons received higher warmth and competence ratings than non-surgeons (*p* = .007). Consistent with gender stereotypes, female surgeons received higher warmth ratings (*p* < .001) and lower competence ratings (*p* = .001) than male surgeons. The stereotype of surgeons held by the general public is that they are high in warmth and competence relative to other occupational groups. Surgeon gender appears to influence general beliefs about the warmth and competence of female and male surgeons.

## Introduction

Patients’ impressions of surgeons play an important role in treatment outcomes. In particular, patients’ impressions of surgeon competence (knowledge, skill, capability) and warmth (goodwill, empathy, beneficence) are associated with greater trust in surgeons,[1, 2] satisfaction with treatment outcomes,[3, 4] post-operative pain and analgesic use,[5] adherence to post-surgical rehabilitation programs,[6] referral patterns,[7] and incidence of malpractice claims.[8] Despite the impact of patients’ impressions of surgeons for treatment outcomes, there is a dearth of research into factors that *shape* patients’ impressions of surgeons’ competence and warmth.

Patients’ impressions of their surgeon’s warmth and competence may be informed by a surgeon’s behavior,[5, 7–10] or by third party testimony.[11] Patients’ impressions of surgeons are also likely to be shaped by social stereotypes. Stereotypes are socially learned and culturally-derived perceptions of the attributes and behavior of group members.[12] They are not always consciously held or in agreement with people’s explicit beliefs,[13] and are not especially accurate, particularly in relation to individual group members.[14, 15] Nevertheless, stereotypes are often relied upon–consciously or unconsciously—as a “short cut” for forming impressions of others, particularly in situations that are time-pressured, stressful or anxiety-provoking.[16–19]

Previous research into stereotypes about surgeons has almost exclusively represented the views of medical students and nursing staff, who perceive surgeons to be egoistic, stubborn, aggressive, competitive, and domineering, but also technically masterful, astute, energetic, and precise.[20–28] In other words, surgeons have been described by medical “insiders” as low in warmth but high in competence. These stereotypes about surgeons are not necessarily generalizable to the general public, however, whose views may be more representative of past, current, or future surgical patients. In addition, previously reported evaluations of surgeons have been relative rather than absolute: surgeons were perceived by medical staff and students to be less nurturing and more hierarchical in comparison to pediatricians and general practitioners,[22] however, they may not be perceived as such in comparison to non-medical professionals, particularly since they are in a healthcare field which indicates some level of care for the well-being of people. Finally, much of the research describing perceptions of surgeons was conducted more than 20 years ago, at a time when the surgeon-patient relationship was more paternalistic and less patient-centered than is advocated in current medical education. In view of this gap in the literature, and the clear potential for stereotypes to influence patients’ impressions of surgeons, the present study investigates current, publicly-held stereotypes of surgeon warmth and competence, and benchmarks these stereotypes against a selection of non-medical occupational groups.

Based on previous research, we expect surgeons, as a professional group, to be perceived as lower in warmth than competence. As members of the healthcare profession, however, we expect surgeons to be higher in both competence and warmth relative to members of non-medical professions (businessmen, politicians, professional athletes). At the same time, we expect that stereotypes of surgeons may vary by surgeon gender in one of two ways. On the one hand, gender stereotypes (that females are higher in warmth than competence, and males are higher in competence than warmth)[29, 30] may have “spillover effects” on judgments of female and male surgeons,[31, 32] such that female surgeons are perceived to be higher in warmth and lower in competence than male surgeons. On the other hand, gender stereotypes may create “backlash effects” on perceptions of female surgeons, whereby female surgeons are evaluated more negatively than male surgeons with respect to *both* warmth and competence, reflecting perceivers discomfort with females occupying traditionally “masculine” professional roles.[33–35]

## Method

### Study site

Ethical approval was obtained by the Free University Amsterdam Human Ethics Review Board (VCWE) of the Faculty of Behavioral and Movement Sciences, Department of Experimental and Applied Psychology, 2013), a member of the National Ethics Council for Social and Behavioural Sciences (www.nethics.nl). As such, the institutional review committee adheres to national and international codes of ethics, including the Declaration of Helsinki (2008).

### Participants

Four hundred and fifteen participants were recruited through an online crowd sourcing website, “Crowdflower”, in 2013, which at the time of this study required members of the online platform to hold a US credit card. Hence, participants in this study were assumed to be US residents. Members were provided with information about the study content and purpose and were required to indicate their consent to participate in research before answering any questions.

Respondents were paid $1.00(USD) to participate in a 3–5 minute anonymous online survey and were included in analyses if they (a) completed at least 90% of the survey items, (b) recorded a survey completion time of *at least* 2.5 minutes, and (c) had not previously participated in the study (as indicated by their recorded IP address).

### Design and procedure

The current study uses a within- and between-subjects experimental design to examine stereotypes of surgeons in general (relative to non-medical professional groups), and to explore stereotypes of female and male surgeons, specifically.

Participants in the study completed a ‘Social Perception Task’, in which they indicated their perceptions of the one of the following target evaluation groups to which they were randomly assigned: ‘Females’, ‘Males’, ‘Surgeons’, ‘Female Surgeons’, or ‘Male Surgeons’. In addition, participants indicated their impressions of four non-medical professional groups: TV Celebrities, Politicians, Elite Athletes, and Inner City Police Offers (order counter-balanced). These additional evaluations served to mask the target evaluation groups and to provide a benchmark against which to interpret participants’ ratings of the target evaluation groups. Participants reported whether or not they had previously experienced surgery, as well as their age, gender, and ethnicity.

### Measures

Participants used a 5-point scale (*1 = not at all*, *2 = slightly*, *3 = moderately*, *4 = very*, *5 = extremely*) to indicate their general perceptions of “the attributes associated with different social groups”. Based on previous research,[36] stereotypes about warmth were gauged by asking participants to indicate the extent to which the members of a social group were ‘warm’ ‘tolerant’, ‘good-natured’, ‘sincere’, ‘friendly’, ‘well-intentioned’, and ‘trustworthy’, and stereotypes about competence were assessed by asking participants to indicate the extent to which the members of a social group were ‘competent’, ‘skilful’, ‘independent’, ‘confident’, ‘competitive’, ‘intelligent’, ‘capable’, and ‘efficient’. The order in which these attributes were presented to participants was randomized and counterbalanced. Mean warmth and competence ratings were calculated for the target and comparison evaluation groups. A scale reliability analysis of warmth items (α = .88) and competence items (α = .89) indicated high internal consistency. All statistical analyses were conducted using SPSS v22.

### Statistical analysis

Mean warmth and competence scores were compared using a paired-samples *t*-test. These stereotypes of surgeon warmth and competence were evaluated against selected non-medical professional groups (TV Celebrities, Inner City Police Officers, Politicians and Elite Athletes) using separate one-way repeated measures ANOVAs for warmth and competence. To examine sex effects, a 5 (Target Group: Surgeons, Male Non-Surgeons, Female Non-Surgeons, Male Surgeons, Female Surgeons) × 2 (participant sex: Male, Female) ANOVA with planned contrasts was conducted separately for warmth and competence ratings. We included previous surgery (dummy-coded) as a covariate. We planned the following between-groups contrasts: (1) Female Non-Surgeons vs. Male Non-Surgeons; (2) Female Surgeons vs. Male Surgeons; (3) Female Surgeons vs. Surgeons (unspecified sex); (4) Male Surgeons vs. Surgeons (unspecified sex); (5) Female Non-Surgeons vs. Female Surgeons; and (6) Male Non-Surgeons vs. Male Surgeons. We examined all interactions and controlled Type I error rate using the Hochberg method, to give α = .01.

## Results

### Sample characteristics

There were 328 participants included in the analysis after applying the exclusion criteria (mean age = 31.5 years, range = 16–75 years, 34.5% female, 65.5% male). Participants identified themselves as Caucasian American (34.6%), Asian or Asian American (34.8%), African American (4.3%), Hispanic or Hispanic American (5.8%), Native American (1.5%), or “Other” (19.0%). Approximately half of the participants (47.6%) had previously had surgery: 35.1% with a male surgeon, 7.9% with a female surgeon, 6.1% with both a male and a female surgeon, and 3.4% did not remember the gender of their surgeon.

### Surgeon stereotypes

Overall, surgeons (unspecified sex) were rated as higher in competence (*M* = 4.13, *SD* = 0.62) than warmth (*M* = 3.49, *SD* = 0.68), *t*(62) = 7.18, *p* < .001.

**Fig 1:**
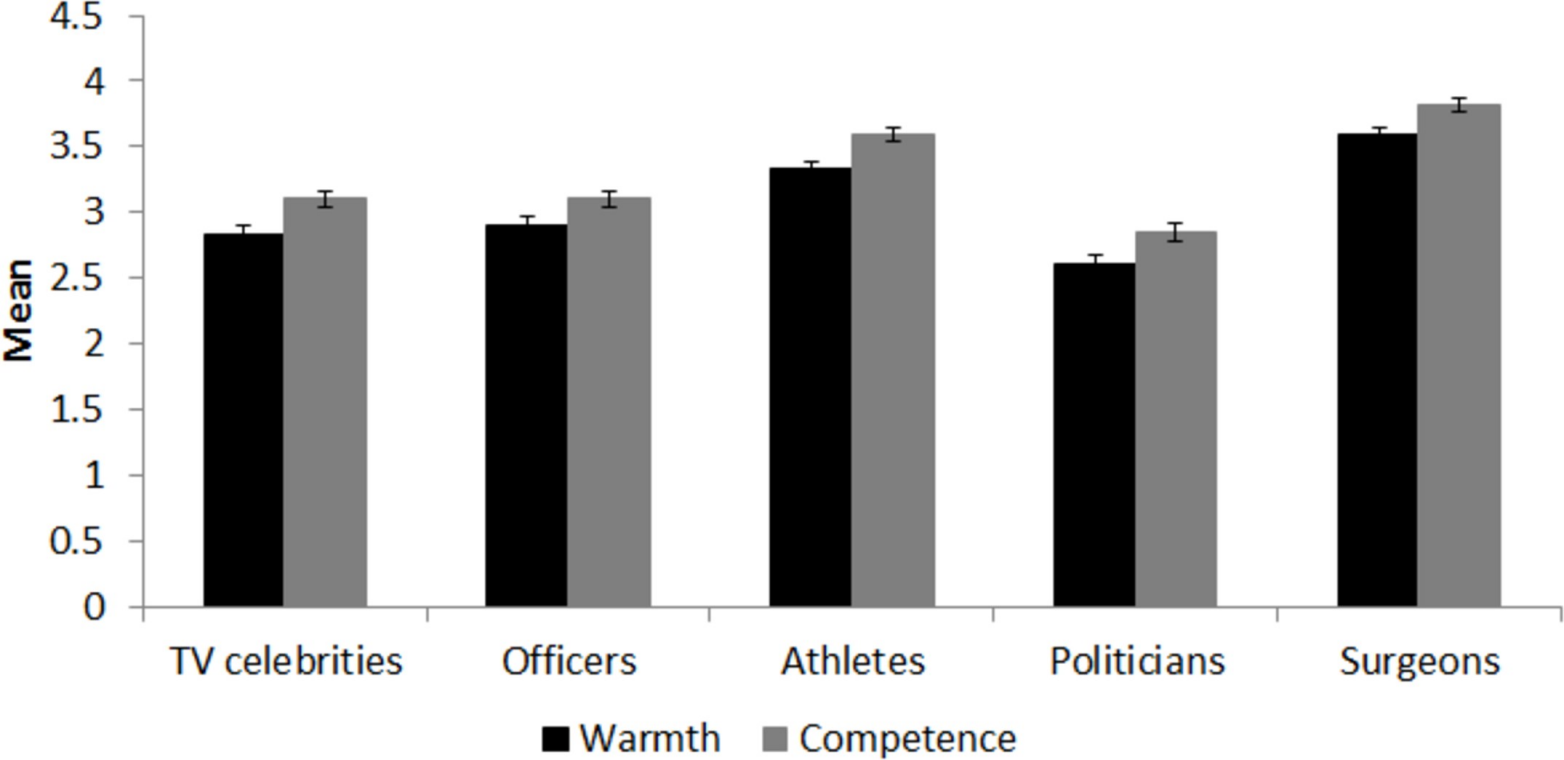
Mean competence and warmth ratings for surgeons and each of the four comparison professional groups.

As shown in Fig 1, Surgeons (unspecified sex) received significantly higher warmth ratings than selected non-medical professional groups, including TV Celebrities, *F*(1,195) = 121.32, *p* < .001, η_p_^2^ = .38, Inner City Police Officers, *F*(1,195) = 125.05, *p* < .001, η_p_^2^ = .39, Politicians, *F*(1,195) = 199.48, *p* < .001, η_p_^2^ = .51, and Elite Athletes, *F*(1,195) = 27.31, *p* < .001, η_p_^2^ = .12. Surgeons (unspecified sex) also received significantly higher competence ratings than TV Celebrities, *F*(1,195) = 118.31, *p* < .001, η_p_^2^ = .38, Inner City Police Officers, *F*(1,195) = 147.28, *p* < .001, η_p_^2^ = .43, Politicians, *F*(1,195) = 211.16, *p* < .001, η_p_^2^ = .52, and Elite Athletes, *F*(1,195) = 23.09, *p* < .001, η_p_^2^ = .11.

### Surgeon gender

#### Warmth ratings

Female Surgeons were rated as higher in warmth than Male Surgeons *F*(1,311) = 22.77, *p* < .001, η_p_^2^ = .07. Female Surgeons were also rated as higher in warmth than Female Non-Surgeons, *F*(1,311) = 12.59, *p* < .001, η_p_^2^ = .04, and Surgeons (unspecified sex), *F*(1,311) = 15.88, *p* < .001, η_p_^2^ = .05.

There was no difference between the warmth ratings given to Surgeons (unspecified sex) and Male Surgeons, *F*(1,311) = 0.38, *p* = .539 η_p_^2^ = .001. Further, there was no difference between the perceived warmth of Male Non-Surgeons and Male Surgeons, *F*(1,311) = 1.45, *p* = .229, η_p_^2^ = .01. Replicating previous research, Female Non-Surgeons were perceived as significantly warmer than Male Non-Surgeons, *F*(1,311) = 7.00, *p* = .009, η_p_^2^ = .02 (see Table 1). None of these effects differed significantly depending on participant sex (i.e., there were no interaction effects). Further, there was no difference in the warmth ratings reported by Female and Male participants overall, *F*(1,311) = 0.50, *p* = .482, η_p_^2^ = .002.

**Table 1:** Warmth and competence means (and standard deviations) for each target group.

Participants	Female non-surgeons	Male non-surgeons	Surgeons	Female surgeons	Male surgeons	Total
***Warmth***	3.46 (0.76)	3.20 (0.61)	3.49 (0.68)	3.93 (0.72)	3.38 (0.72)	3.49 (0.74)
***Competence***	3.53 (0.74)	3.58 (0.64)	4.13 (0.62)	3.50 (0.56)	3.85 (0.75)	3.72 (0.70)

#### Competence ratings

Male Surgeons were perceived as significantly more competent than Female Surgeons, *F*(1,311) = 10.58, *p* = .001, η_p_^2^ = .03 (see Table 1). Surgeons (unspecified sex) received significantly higher competence ratings than Female Surgeons, *F*(1,311) = 30.35, *p* < .001, η_p_^2^ = .09, and there was no difference in evaluations of the competence of Female Surgeons and Female Non-Surgeons, *F*(1,311) = 0.66, *p* = .416.When applying the Bonferroni correction, ratings of Surgeons (unspecified sex) were not significantly different from ratings of Male Surgeons, *F*(1,311) = 5.59, *p* = .019, η_p_^2^ = .02. However, Male Surgeons were rated as significantly more competent than Male Non-Surgeons, *F*(1,311) = 6.90, *p* = .009, η_p_^2^ = .02. In contrast to previous research, there was no difference between the rated competence of Female and Male Non-Surgeons, *F*(1,311) = 0.02, *p* = .895, η_p_^2^ < .001. None of these effects interacted with participant sex. Further, the competence ratings reported by Female and Male participants were not statistically different, *F*(1,311) = 3.57, *p* = .060, η_p_^2^ = .01.

## Discussion

In contrast to the view that surgeons are low in social skills but high in technical skills, participants from the general population (48% of whom had previously been a surgical patient) rated surgeons, as a group, as high in warmth as well as competence, relative to other non-medical occupational groups. In addition, the extent to which surgeons were rated as high in warmth and competence depended on their gender: As a group, female surgeons received higher warmth ratings than male surgeons, while male surgeons received higher competence ratings than female surgeons. Our findings did not reveal evidence for “backlash effects”, whereby females who occupy traditionally male-dominated professions (i.e. surgery) are perceived as less warm than females in general.[34] Instead, female surgeons were perceived to be higher in warmth than both male surgeons and females in general.

It is important to emphasize that the stereotypes reflect perceptions, not reality:[28] Female surgeons, as a group, are not necessarily more compassionate and caring than male surgeons, and male surgeons, as a group, are not necessarily more skilled and capable than female surgeons. There is evidence that female general practitioners exhibit more empathic behavior than male general practitioners.[37–39] However, this finding more likely reflects differences in the way that patients communicate with female and male doctors (disclosing greater emotion and need for empathy to female compared to male doctors), rather than differences in the way that female and male physicians communicate in general.[40] Patient behavior towards female and male *surgeons* has not been investigated, however in light of our finding that people expect female surgeons to be higher in warmth than male surgeons, we would expect patients to show more partnership building, question asking, social conversation, and emotional disclosure with female surgeons compared to male surgeons. I may be because of patients’ greater willingness to communicate more demonstratively with female surgeons that female surgeons then display greater warmth than male surgeons.

It important to acknowledge that there will be conditions under which stereotypes are not used to form judgments of surgeons in a clinical context. Stereotypes are generally not used to form impressions of others when their attributes are unambiguous or if their attributes have been explicitly described by a reliable source.[11] For example, if a male surgeon’s communication behavior is unambiguously warm, or if a patient has been referred to a particular male surgeon because of his exceedingly compassionate and caring bedside manner, then his gender is less likely to undermine patients’ perceptions of his warmth relative to a female surgeon. In the same vein, if patients have been recommended to a female surgeon because “she is the best and most trusted surgeon”, her competence is less likely to be undervalued relative to a male surgeon.

Nevertheless, the finding that people hold positive stereotypes of surgeons (both male and female) bodes well for perceptions of surgeon warmth and competence in interactions with patients. Stereotypes have been shown to shape judgments of behavior in two ways, both by increasing peoples’ attention to and memory for stereotype consistent behavior, and by influencing the interpretation of ambiguous behavior.[18, 41] Theoretically, therefore, the stereotype of surgeons as both competent and warm may lead patients to interpret paternalistic communication (“do as I say and you will be healed”) as a sign of confidence and concern for patient well-being rather than arrogance and a lack of empathic concern. Again, this is a fertile area for future research.

### Strengths and limitations

Participants in the current study reported their perceptions of surgeons (unspecified sex), male surgeons, or female surgeons, in response to an online survey. There are two main strengths to this research design and procedure. Online surveys enable researchers to recruit a participant sample that is heterogeneous in terms of age, ethnicity and education. Consequently the social attitudes that are reported by participant samples recruited online tend to be more reliable (generalizable) than reports provided by selective samples of medical or psychology students.[42] An additional strength of this research method is that it is “double-blind”, in the sense that researchers and participants do not come face to face with each other. Hence, the social attitudes reported by participants in the present study are unlikely to be biased by participants’ social desirability concerns, or the gender of the researcher conducting the study.

Several researchers have independently validated the use of online participant recruitment for the collection of reliable (generalizable) data on publicly held opinions.[42–44] However, the opinions reported by participants in the current study are likely to be specific to residents of the USA, and may also be specific to members of online survey distribution platforms such as Crowdflower (used in the present study), or Mechanical Turk (Amazon). It has been argued that persons who are recruited to participate in online surveys and experiments through crowsourcing websites such as these may in some cases be “non-naïve”, having participated in many similar studies over time[45]. If researchers post various iterations of the same study on the same recruitment website, it is certainly possible that participants would become familiar with the type of questions being asked and may even get a sense of what the experimenter is investigating, leading to demand effects. The present study recruited participants for a single study in one iteration, and hence non-naivety of participants is unlikely to a concern in the present research.

Nevertheless, given the experimental nature of the present research we would caution against making inferences about how patients perceive and respond to female and male surgeons based on the current data alone. Follow-up research studies are required to observe how these stereotypes influence patients’ impressions of surgeons in a clinical context. On the one hand, interactions between patients and surgeons are relatively limited, especially in comparison to primary care specialists. Hence, it is plausible that stereotypes of surgeons (both female and male) predict patients’ impressions of surgeons in practice. On the other hand, research suggests that when people are motivated to form accurate impressions of others (as one might expect when a patient is *choosing* a surgeon), they tend to use individuating information to form impressions rather than stereotypes.[41] Hence, in the context of elective surgery, patients may have the opportunity to “interview” their surgeon and may be less likely to use stereotypes about surgeons (global or gender-specific) when evaluating the warmth and competence of their prospective surgeon. In the context of emergency surgery, however, or in cases where the patient does not have an opportunity to even meet their surgeon, patients may be more likely to rely on their internalized beliefs or stereotypes about female and male surgeons to form their impressions.

### Future directions

The current research suggests that there is a general held perception that surgeons, as a group, are higher in warmth and competence than other professional groups and that female surgeons, specifically, are perceived to be higher in warmth and lower competence than male surgeons. In consideration of the limitations of the current research, described above, the implications of the current results are unclear. In particular, it will be important for future research to investigate whether social stereotypes of male and female surgeon attributes bias *patients’* expectations and impressions of their surgeons. Such studies will by necessity be observational in nature, and may benefit from using implicit (behavioral) measures of patient preferences, such as waitlist demands on male versus female surgeons for elective surgeries, rather than patient self-reported preferences or behavioral intentions. In many countries, such as the United States, patients provide satisfaction ratings (Press Ganey Scores) which may provide insight into whether patients’ expectations of differences in male and female surgeon warmth and competence have any consequences for their experience of female and male surgeon warmth and competence. It will be interesting to note whether there are differences between patients’ explicitly reported satisfaction ratings of male and female surgeons following non-elective (emergent) surgeries. Finally, it will be important to consider factors that moderate patients’ expectations of and preferences for surgeon attributes. For example, the degree to which patients expect and value surgeon warmth may depend on the type of surgery being performed (e.g. mastectomy vs. lung transplant)[11], or whether the surgery is elective (e.g. cosmetic surgery), potentially life-saving surgery (e.g. prophylactic cardiac surgery) or emergent surgery.

## Conclusion

The current study contributes to our understanding of patient expectations and impressions of surgeons by examining generally held beliefs or stereotypes about surgeons, as a professional group. The results of this study suggest that surgeons are, as a group, expected to be high in both warmth and competence relative to other professional groups and that gender stereotypes may “spillover” to further nuance beliefs and expectations of female and male surgeons. In consideration of the experimental methodology used in the current research, additional studies are recommended to validate the generalizability of the current findings with a patient population.

## Supporting information

S1 File Supplementary material data file. (XLSX)
